# Clinical study on swallowing function of brainstem stroke by tDCS

**DOI:** 10.1007/s10072-021-05247-6

**Published:** 2021-05-11

**Authors:** Huiwen Mao, Yi Lyu, Yan Li, Lin Gan, Jiawei Ni, Liang Liu, Zhengguang Xiao

**Affiliations:** 1 Department of Rehabilitation Medicine, Tongren Hospital, Shanghai Jiao Tong University School of Medicine, 1111 Xianxia Road, Shanghai, 200336 China; 2 Department of Anesthesiology, Minhang Hospital, Fudan University, Shanghai, 201100 China; 3 Department of Medical Imaging, Tongren Hospital, Shanghai Jiao Tong University School of Medicine, 1111 Xianxia Road, Shanghai, 200336 China

**Keywords:** Brainstem stroke, Transcranial direct current stimulation, Dysphagia, Nutrition, Infection

## Abstract

**Objective:**

To investigate the effect of transcranial direct current stimulation (tDCS) combined with conventional comprehensive rehabilitation on dysphagia after brainstem stroke.

**Materials and methods:**

Forty brainstem stroke patients were randomly divided into tDCS group and conventional comprehensive treatment group, including 20 patients in each group. Both groups were given routine swallowing function training, and tDCS group added transcranial direct current stimulation (tDCS). The Dysphagia Outcome and Severity Scale (DOSS) and Functional Dysphagia Scale (FDS) were evaluated respectively before and after 8 weeks of continuous treatment with VFSS. The white blood cell (WBC), c-reactive protein, prealbumin (PAB), albumin (Alb), and hemoglobin (Hb) were also compared between the two groups before and after 8 weeks of continuous treatment.

**Results:**

After 8 consecutive weeks of treatment, the score of DOSS scale and FDS scale in both groups was improved (*P* < 0.05), WBC and CRP were decreased (*P* < 0.05), and Alb and Hb were improved (*P* < 0.05), and PAB had no differences (*P*=0.474). The tDCS group was superior to conventional comprehensive group in improving the swallowing function and nutritional indexes (*P* < 0.05).

**Conclusions:**

tDCS therapy combined with routine training can improve the swallowing function and nutritional status of patients, and reduce infection.

## Introduction

Brainstem stroke accounts for about 9 to 21.9% of stroke [1]. There is a control center of swallowing in the brainstem, such as ambiguous nucleus, solitary tract nucleus, and surrounding reticular structure. Therefore, stroke in the medulla oblongata is called true bulbar paralysis, whose incidence is as high as 51 to 100% [2, 3]. The characteristics of main performance for pharyngeal swallowing disorder such as prolonged food passage in pharyngeal period, food residues in epiglottis and piriform fossa, abnormal upper laryngeal lift, and delayed ring pharyngeal muscle. Dysphagia will increase the incidence of complications such as dehydration, aspiration, pneumonia, and malnutrition, which not only affects the quality of life of patients but also increases the mortality and prolongs the duration of rehabilitation and hospitalization. It takes long time to recover and brings heavy economic burden to the family and the whole society [4, 5].

Several interventions such as cricopharyngeal muscle myotomy, injection of botulinum toxin A, catheter balloon dilatation, and conventional dysphagia treatments have been recommended for brainstem stroke patients with dysphagia. While these methods target the peripheral nervous system. Transcranial direct current stimulation (tDCS), as a non-invasive brain stimulation (NIBS) technique, has shown increasing evidence remodeling the brain network to improve the motor, cognitive, and speech functions [6, 7]. Recent years, there have been a few studies show that stimulation over the cortical swallowing center can improve swallowing function, while these strokes occurs in the cortex [8, 9].The effect of such a combined strategy remains unknown on the swallowing function in brainstem stroke patients. In our study, tDCS therapy was applied to the brainstem stroke patients with dysphagia combined with conventional dysphagia rehabilitation to observe the effect of this technique. We aim to explore a more effective and safer rehabilitation training program for dysphagia after brainstem stroke.

## Materials and methods

### Study population

**Fig. 1:**
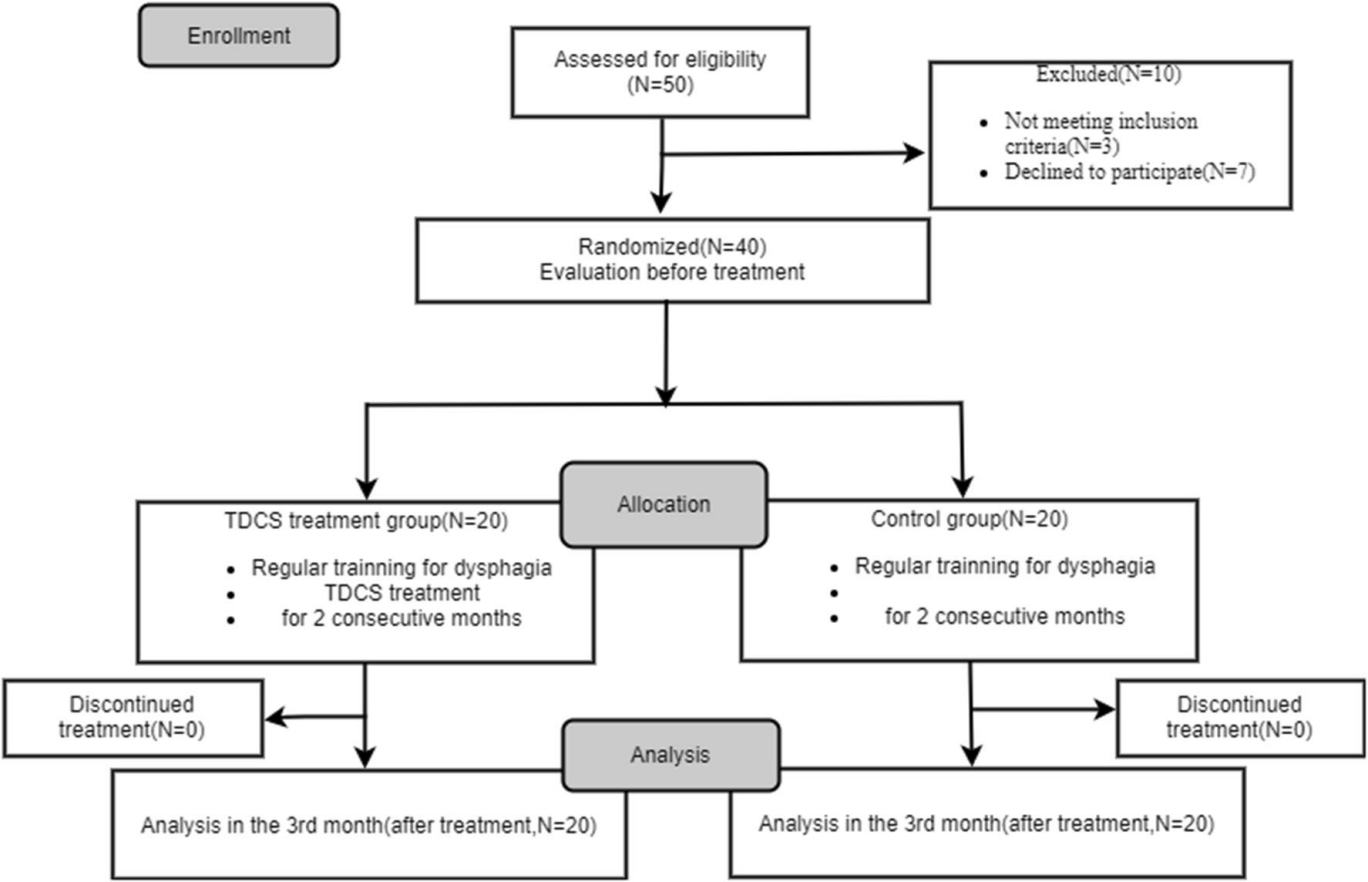
Enrollment flow chart

Our study has been approved by the Ethics Committee of Tongren Hospital, Shanghai Jiao Tong University School of Medicine (2017-009-01), including 50 patients from October 2017 to December 2018 in our Rehabilitation ward. Ten of them were excluded (3 not meeting inclusion criteria and 7 declined to participate). These patients were randomly allocated into tDCS group and control group, with 20 patients in each group by a computer-generated randomization list. All met the inclusion criteria below. All assessments in both groups were performed by a certain therapist, who did not treat these enrolled patients and also blinded to the treatment allocation. These patients have not been in any study before our study’s enrolment. Patients in both groups were given secondary prevention of stroke: (1) Control of hypertension, blood pressure should be controlled below 140/90mmHg, hypotensive speed, amplitude, specific use of drugs should be individualized, to avoid hypoperfusion. (2) Control of diabetes, lifestyle and drug intervention, the control of HbA1C<7%. (3) For patients with non-cardiogenic embolic ischemic stroke, antiplatelet drugs, aspirin 100mg/day, or clopidogrel 75mg/day were given, and for patients with cardiogenic embolic stroke, oral warfarin was given, and the target dose was maintained at the INR level of 2.0–3.0. (4) Management of lipid metabolism, the use of statins to control LDL-C ≤1.8mmol/L. The enrollment flow diagram is illustrated in Fig. 1.

### Inclusion criteria

(1) The brainstem stroke was diagnosed according to the World Health Organization’s definition of a stroke, with the location on the medulla oblongata confirmed by CT or MRI, with the symptom of water choking, dysphagia, and hoarseness; (2) Age from 50 to 80 years old; (3) The course of the disease from 2 to 12 months; (4) NIHSS score from 2 to 9; (5) No cognitive impairment, good compliance; (6) Signing informed consent; can tolerate our therapy.

### Exclusion criteria

(1) The stroke site was not located in the brainstem; (2) Other diseases of the nervous system caused swallowing disorders; (3) Head and neck radiotherapy caused swallowing disorders; (4) Critical conditions such as organ failure or unconsciousness; (5) dysphagia due to previous neurological dysfunction. 

### Experimental procedures

**Fig. 2:**
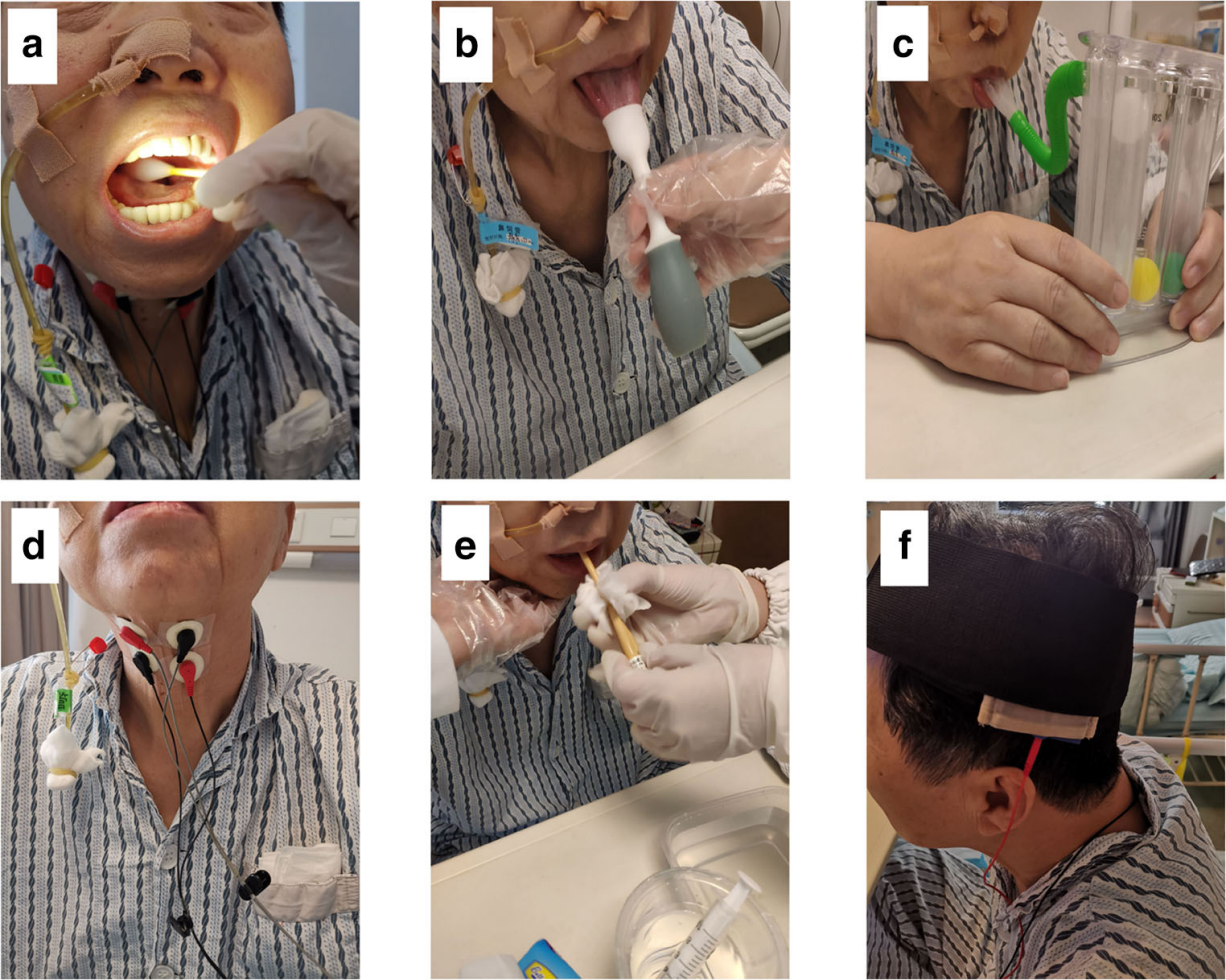
The tDCS group cooperated with tDCS training on the basis of routine training. **a** Iced cotton swab stimulates the buccal and pharyngeal. **b** Tongue muscle extension. **c** Breathing training. **d** Pharyngeal electrical stimulation. **e** Swallowing balloon expansion treatment. **f** tDCS training

The conventional comprehensive treatment group received routine swallowing rehabilitation training. The tDCS group cooperated with tDCS training based on routine training (Fig. 2).

Specific methods are as follows:

1. Routine swallowing rehabilitation training:
  - Motor and sensory training of the muscles around the mouth and tongue, such as lip closure, mouth opening, sucking, and tongue extension. Using iced cotton swab to stimulate the pharyngeal posterior wall and soft palate mucosa 60min each time, once a day, 6days per week, a total of 8 weeks.
  - Breath training: teaching the patient to take abdominal breath, slowly exhale the air with a breathing trainer, and airway protection strategies, once a day, 6days per week, a total of 8 weeks.
  - External laryngeal electrical stimulation: Vital Stim neuromuscular electrical stimulation instrument was used for treatment, and body surface electrodes were placed on the skin of pharynx, once a day, 6days per week, a total of 8 weeks.
  - Balloon dilatation [10]: using No. 12 catheter inserted through the mouth slowly into the esophagus, inject 2–8 mL cold saline slowly into the balloon until the patients feel stuck, then instruct the patient to swallow while gently pulling catheter outward, once feeling the resistance of balloon has passed the cricopharyngeus muscle, quickly draw out the saline in the balloon catheter. Eight times a day, 6days per week, a total of 8 weeks.
2. tDCS training protocol:

tDCS training was delivered by a constant current stimulator (IS200, Sichuan Intelligent Electronics Industry Co., Ltd.) through 2 saline-soaked electrodes. The anodal electrode with the size of 4.5cm*6.0cm was placed over the swallowing sensory motor cortex area on the non-lesional hemisphere, at the middle of C3 and T3 according to the international 10-20 EEG electrode system, and the cathodal electrode with the size of 7cm*10.0cm was placed on the opposite shoulder. The current was 1.6mA, 20 min each time, once a day, 6days each week, for a total of 8 weeks.

### Outcome measurement

#### Dysphagia Outcome and Severity Scale (DOSS)

DOSS scale is Deglutition function was divided into 7 levels according to the degree of damage, with 1 point representing severe dysphagia: there were many oral or pharyngeal residues that could not be transported by the tongue and muscle in oral cavity, no functional autonomic cough, aspiration, or inability to complete deglutition. A score of 6 and 7 represents normal swallowing, a score of 5 represents mild dysphagia, a score of 2 to 4 represents moderate dysphagia, and a score of 1 represents severe dysphagia [32].We evaluate the score by taking video-fluoroscopic swallowing study. The score changed to 5 and 6 after treatment, and we define it as effective.

#### Functional Dysphagia Scale (FDS)

Used to evaluate oral and pharyngeal swallowing function, based on swallowing fluoroscopy Video imaging examination (video fluoroscopic swallowing study, VFSS) can see the objective of image data and is composed of 11 items: food group formation, in the mouth, lips closed and food residue, oral transportation time, start, throat swallowing up, closed epiglottis, nasal regurgitation, epiglottis valley food residue, pharynx posterior wall after swallowing food residue, pharynx ministry through time [8, 33]. We evaluate the score by taking video fluoroscopic swallowing study.

#### Video fluoroscopic swallowing study

Using Shimazu large plate multifunctional digital perspective photography system, patients sat on the chair before the machine, and patients were asked to swallow 4 portions of standardized formula (5mL each thin, thick, paste and water) containing barium sulfate. The swallowing process was recorded which can clearly show swallowing organs and its structure. All VFSS images were reviewed by an experienced radiologist. The radiologist was blinded to the clinical information.

All VFSS images were reviewed by an experienced radiologist (Zhengguang Xiao) based on the FDS scale. The radiologist was blinded to the clinical information.

#### Nutrition indicators

Hemoglobin (Hb), albumin (Alb), and prealbumin (PAB) were determined.

#### Infection indicators

White blood cell (WBC) and c-reactive protein (CRP) were determined.

### Statistical analysis

All data was analyzed by SPSS 22.0 software (IBM, Inc.). FDS, PAB, Alb, Hb, WBC, and CRP results before and after treatment between the two groups were compared by an independent sample *t*-test and paired *t*-test within each group. DOSS results were compared by chi-square. Statistical significance was determined when *P*<0.05.

## Results

### General information

There were 11 males and 9 females in the tDCS group. The age was 59.8±7.3 years old, the disease course was 3.3±2.2 months, and the NIHSS score was 5.3±3.2, among which 18 patients had basic diseases such as hypertension, 15 patients had diabetes, 10 patients had coronary heart disease, and 15 patients had hyperlipidemia. In the conventional comprehensive treatment group, there were 8 males and 12 females, with an age of 61.3±8.0 years old, a course of disease of 3.6±2.5 months, and a NIHSS score of 5.7±2.8, among which there were 16 cases of basic diseases such as hypertension, 12 cases of diabetes, 8 cases of coronary heart disease, and 16 cases of hyperlipidemia. The stroke location of group tDCS includes medulla 5 patients, midbrain 8 patients, and pons 7 patients. The stroke location in control group includes medulla 4 patients, midbrain 9 patients, and pons 7 patients. The baseline data of the two groups have no significant difference (*P* > 0.05) (Table 1).

**Table 1:** Clinical baseline information of participants (gender, age, clinical course, NIHSS score, stroke type) (Mean±SD)

Group	*n*	Gender		Age (year)	Clinical course (month)	NIHSS score	Stroke type		Location		
		M	F				Infarct	Hemorrhage	Medulla	Midbrain	Pons
tDCS group	20	11	9	59.800±7.267	3.250±2.243	5.250±3.150	20	0	5	8	7
Control group	20	8	12	61.250±8.022	3.600.±2.494	5.650±2.817	18	2	4	9	7
*P* value				0.467	0.571	0.607					
*T* value				0.734	0.571	0.5183					

The two groups were compared using unpaired *t*-tests

### Effects compared between the two groups

After each group received 8 consecutive weeks of treatment, the results were as follows:

#### Comparison of results before and after treatment in the tDCS group

For the evaluation of swallowing function, FDS score before and after treatment was 75.4±12.4, and 46.2±18.8 (*P* < 0.001), which was improved compared with that before treatment. The effective rate of DOSS was 45%. In terms of nutritional indicators, ALB was 35.5±1.0 before treatment and 52.9±3.0 after treatment (*P* < 0.001), and Hb was 113.6±1.7 before treatment and 148.4±0.6 after treatment (*P* < 0.001), all of which were improved compared with that before treatment, PAB was 213.3±18.6 before treatment and 254.3±21.3 after treatment (*P* =0.155), showing no significant difference compared with that before treatment. In terms of infection indicators, blood WBC was 11.3±11.6 before treatment and 3.9±3.1 after treatment (*P* =0.002).CRP was 30.9±9.9 before treatment and 5.5±1.0 after treatment (*P*=0.0152), both of which were better than that before treatment (Table 2).

**Table 2:** Clinical baseline information of participants before and after treatment (FDS)

Group	FDS	
	Before	After
tDCS group (*n*=20)	75.400±12.370	46.150±18.821
Control group (*n*=20)	75.300±12.507	56.750±13.756
*P* value	0.975	0.0173
*T* value	0.031	2.490
95% confidence interval	(−6.405, 6.605)	(−19.220, −1.979)

The two groups were compared unpaired *t*-tests

#### Comparison of results before and after treatment in the conventional comprehensive treatment group

For the evaluation of swallowing function, FDS before and after treatment was 75.3±12.5 and 56.8±13.8 (*P* < 0.001), which was improved compared with that before treatment. The effective rate of DOSS was 10% (Table 3). In terms of nutritional indicators, ALB was 35.6±2.1 before treatment, 44.8±0.9 after treatment (*P* =0.004), 112.5±1.9 before treatment, and 129.3±2.2 after treatment (*P* < 0.001), all of which were improved compared with that before treatment. There were no significant differences between PAB before treatment and 234±18.4 (*P* =0.064) after treatment. After treatment, it was 5.9±3.2 (*P*= 0.005), CRP was 32.9±7.7 before treatment, and CRP was 10.1±1.8 after treatment (*P*=0.007) (Table 4).

**Table 3:** Clinical information of participants after treatment (DOSS)

Group	DOSS		
	Effective	Invalid	Total effective rate (%)
tDCS group (*n*=20)	9	11	45
Control group (*n*=20)	2	18	10
*X*^2^			6.144
*P* value			0.0132

The two groups were compared chi-square test

**Table 4:** Clinical information of participants before and after treatment (WBC,CRP)

Group	WBC		CRP	
	Before	After	Before	After
tDCS group (*n*=20)	11.290±11.609	3.989±3.089	30.980±9.899	5.475±0.965
Control group (*n*=20)	11.690±9.994	5.999±3.177	32.940±7.712	10.110±1.821
*P* value	0.887	0.018	0.877	0.031
*T* value	0.143	2.485	0.156	2.247
95% confidence interval	(−6.065, 5.265)	(−3.649, −0.372)	(−27.52, 23.59)	(−8.806, −0.458)

#### Comparison between the two groups after treatment

In terms of swallowing function, FDS were 46.2±18.8 and 56.8±13.8 (*P*=0.0173), respectively. The effective rate of DOSS was 45% and 10% (*P*=0.0132, *X*^2^=6.144). In terms of nutritional indexes, ALB was 52.9±3.0 and 44.8±0.9 (*P*= 0.035) after treatment, and Hb was 148.4±0.6 and 129.3±2.2 (*P* < 0.001), respectively. The tDCS group was superior to the conventional comprehensive treatment group, and PAB was 254.3±21.3 and 234.0±18.4 (*P*=0.474), respectively. In terms of infection indicators, blood WBC was 3.9±3.1 and 5.9±3.2 (*P*= 0.018), and CRP was 5.5±0.9 and 10.1±1.8 (*P*=0.031), respectively. The tDCS group was superior to the conventional comprehensive treatment group (Table 5).

**Table 5:** Clinical information of participants before and after treatment (prealbumin, albumin, hemoglobin)

Group	Prealbumin (PAB)		Albumin (Alb)		Hemoglobin (Hb)	
	Before	After	Before	After	Before	After
tDCS group (*n*=20)	213.300±18.610	254.300±21.330	35.500±1.030	52.850±3.039	113.600±1.658	148.400±0.573
Control group (*n*=20)	188.700±14.990	234.000±18.370	35.550±2.112	44.750±0.890	112.500±1.931	129.300±2.163
*P* value	0.310	0.474	0.035	0.035	0.682	<0.001
*T* value	1.030	0.723	0.033	2.189	0.413	8.513
95% confidence interval	(−23.790, 72.990)	(−36.670, 77.370)	(−3.079, 2.979)	(0.605, 15.590)	(−4.105, 6.205)	(14.52, 23.58)

The two groups were compared using unpaired *t*-tests

## Discussion

The brainstem of swallowing disorder after stroke is often defined as the true bulbar paralysis with the incidence of 51–100% [11–14]. It takes long time to recover. The characteristics of main performance for pharyngeal swallowing disorder such as prolonged food passage in pharyngeal period, food residues in epiglottis and piriform fossa, abnormal upper laryngeal lift, and delayed ring pharyngeal muscle. It will lead to impaired effectiveness and safety of swallowing, ultimately increase the risk of aspiration, aspiration pneumonia, asphyxia, and malnutrition [15, 16]. And the malnutrition is a common finding after stroke. Our study aimed to describe the improvement of malnutrition in stroke patients after our conventional treatments.

Studies have shown that the current rehabilitation treatments for dysphagia after stroke mainly include acupuncture, oral sensation and exercise training, neuromuscular electrical stimulation, balloon dilation technique, mirror therapy, and hyperbaric oxygen [17–26]. These methods can promote the recovery of swallowing function by improving the amplitude of hyoid laryngeal complex lifting or increasing peripheral sensory input to the central nervous system. In recent years, non-invasive brain stimulation techniques such as tDCS have increasingly been used to promote brain function by regulating neurotransmitters, thereby making synapses malleable and ultimately strengthening brain networks. Kumar [27–29] found that tDCS could improve the swallowing function of patients with ataxic dysphagia, and Shigematsu T found that 20min and 1-mA anode stimulation on the affected side could significantly improve the DOSS score of patients after intervention [28]. However, these studies focused on swallowing disorders caused by pseudobulbar palsy, and there were less reports on true bulbar paralysis after brainstem stroke. Furthermore, we selected patients with prolonged dysphagia for more than 2 months.

Our study combined the brain stimulation with conventional swallowing training. We applied tDCS to patients’ contralateral sensorimotor cortex and found that the tDCS group were better DOSS and FDS score than the control group. We also found that tDCS treatment was better than the control group in Alb, Hb, WBC, and CRP, indicating that the treatment improved the swallowing ability and resumed oral feeding, improving the nutritional status, and reducing the infection as well. It may stimulate and activate the subcortical swallowing center by tDCS. The subcortical swallowing center is mainly connected with the cortex of primary sensory motor cortex, premotor cortex, insula, anterior cingulate gyrus, basal ganglia and other parts, and subcortical nerve fibers. We also found that balloon swallowing treatments may induce the swallowing reflex by repeating dilation the pharyngeous muscle. We think the balloon swallowing treatments may increase the range of motion of the cricopharyngeus muscle and improve UES relaxation. Swallowing is a complex motor process. These events are controlled by the peripheral nervous system (PNS) and the central nervous system (CNS). Swallowing occurs in association with the cortex and subcortical regions, brainstem, and peripheral structures. The basic motor strategy for swallowing is generated by the brainstem regarded as CNS. Afferent fibers from PNS transmit sensory information from the oral cavity, larynx and pharynx converge in the nucleus tractus solitarius (NTS), in the medulla. In addition, NTS receives visceral sensory information from CN V in the pons [30, 31]. Increased sensory input to the larynx activates the cortical-brainstem swallowing pathway, which accelerates swallowing initiation and finally reduces aspiration.

The swallowing is initiated by the bilateral centers of the brain. The corticobulbar tracts descend bilaterally to the pons and medulla with bilateral centers that control the muscles of swallowing. In brainstem stroke patients, not only the deglutition reflex center is damaged but also the sensorimotor circuit may have some muscular dyskinesia. Due to the defected function of corresponding area on the damaged hemisphere, swallowing function of the contralateral hemisphere reshape became the basis of swallowing disorder in patients’ recovery. The stimulation of damage of cerebral hemisphere exists the possibility of secondary epilepsy [32]; for security reasons, we choose the contralateral hemisphere to stimulate. The results showed that improving the excitability of the uninjured side swallowing cortex could improve the swallowing function of patients as well as improve the nutritional function of brainstem stroke patients without adverse consequences. When neurologic deficits improve, patients’ nutrition also improves. The result is consistent with the previous research results of some scholars that the stimulation of excitatory tDCS on the uninjured side combined with routine swallowing rehabilitation training could better improve the swallowing disorders in brainstem stroke patients.

This study is limited in lack of sham comparator and quantitative objective indicator. In our future studies should further add functional magnetic resonance and MEP to objectively evaluate the improvement of swallow function. We also need to add a shame comparator group.

## Conclusion

Finally, in future studies, patients should be followed up as long as possible to further explore the neuroregulatory mechanism of swallowing, so as to better improve the dysphagia after stroke. Future studies will appropriately expand the sample size, to further conduct subcomponent types of stroke types and specific parts of brainstem.

## Data Availability

All original data will be available when contact the corresponding author.
